# Impact of deceased-donor characteristics on early graft function: outcome of kidney donor pairs accepted for transplantation

**DOI:** 10.3389/fimmu.2024.1303746

**Published:** 2024-10-08

**Authors:** Christoph F. Mahler, Felix Friedl, Christian Nusshag, Claudius Speer, Louise Benning, Daniel Göth, Matthias Schaier, Claudia Sommerer, Markus Mieth, Arianeb Mehrabi, Lutz Renders, Uwe Heemann, Markus Krautter, Vedat Schwenger, Fabian Echterdiek, Martin Zeier, Christian Morath, Florian Kälble

**Affiliations:** ^1^ Department of Nephrology, University Hospital Heidelberg, Heidelberg, Germany; ^2^ Department of General, Visceral and Transplantation Surgery, University Hospital Heidelberg, Heidelberg, Germany; ^3^ Department of Nephrology, Technical University of Munich, Munich, Germany; ^4^ Department of Nephrology, Hospital Stuttgart, Stuttgart, Germany

**Keywords:** immunology, transplantation - kidney, transplantation, deceased donation, scores

## Abstract

**Introduction:**

The impact of deceased donor characteristics on kidney transplant outcomes is controversial. Correspondingly, the predictive performance of deceased donor scores remains moderate, and many transplant centers lack validated criteria for graft acceptance decisions. To better dissect donor-related risk from recipient and periprocedural variables, we analyzed outcomes of kidney donor pairs transplanted in different individuals.

**Methods:**

This study explored (a)symmetry of early outcomes of 328 cadaveric kidney transplant recipients from 164 donor pairs transplanted at three Eurotransplant centers. The primary discriminatory factor was (a)symmetry of partner graft function, defined as early graft loss or impaired graft function [estimated glomerular filtration rate (eGFR) <30 mL/min] 3 months after transplantation. We reasoned that a relevant impact of donor factors would result in a high concordance rate of limited graft function or failure.

**Results:**

The observed number of symmetric graft failure after transplantation was less than statistically expected (3 months: 1 versus 2, p = 0.89; and 12 months: 3 versus 5, p = 0.26). However, we found a trend toward an impaired 5-year graft survival of grafts with good function 3 months after transplantation but a failed or impaired partner graft compared to symmetrically well-functioning grafts (p = 0.09). Subsequently, we explored the impact of individual donor and recipient variables on early transplant outcomes. Generalized estimating equations after feature selection with LassoGEE bootstrap selected donor age, donor body mass index, and donor eGFR as the relevant risk factors.

**Discussion:**

Our findings indicate that donor factors impact early outcomes in kidney transplantation but may have a limited role in long-term graft survival, once a graft has been accepted for transplantation. Utilizing donor-based clinical scores has the potential to aid clinicians in acceptance decisions, giving them an estimate of individual posttransplant outcomes. However, the ultimate decision for acceptance should rest with clinicians, who must consider the complex interplay of donor factors, as well as recipient and periprocedural characteristics.

## Introduction

Kidney transplantation is the gold-standard therapy for patients with end-stage kidney disease (1). However, clinicians face a difficult decision-making process during organ acceptance, aggravated by a scarcity of optimal donor organs. Development of organ acceptance algorithms to improve individual allocation strategies remains a challenge (2, 3). Different strategies are applied; however, the organ acceptance decisions remain center-specific without universal evidence–based criteria. Applying scoring systems, such as the kidney donor risk index/kidney donor profile index (KDRI/KDPI), has been suggested widely, but its validity in European cohorts is debated. This score calculates the relative risk of individual graft failure compared to a reference donor profile. However, individual donation and procurement factors as well as donor-recipient interactions (e.g., immunological) are omitted (4–6). Beyond these limitations, possibly misleading clinicians within the organ acceptance process, the index interpretation scaled from 0 to 100 is not intuitive and hard to illustrate for patients in a shared decision-making process. In addition to the restrictions of currently used scoring systems, there remains a debate about the general meaning of donor factors. Hence, in this study, we analyzed the outcome of 164 kidney donor pairs transplanted at our center in Heidelberg, at the transplant center of the Technical University in Munich and in Stuttgart. We used kidney function 3 months after transplantation [estimated glomerular filtration rate (eGFR) ≤30 mL/min or early graft loss (EGL) versus eGFR > 30 mL/min] as the discriminatory parameter to select groups and chose graft survival as the primary outcome. We hypothesized that a relevant impact of donor factors would result in (i) an increased rate of symmetric graft failure and (ii) a 5-year survival benefit in pairs with symmetrically good graft function 3 months after transplantation compared to index grafts with good function but an impaired or failed partner graft function (asymmetric early outcome).

## Materials and methods

### Study cohort

In this study, we retrospectively included N = 1,353 deceased kidney donor transplantations between 2006 and 2021 at our center (605 grafts), Stuttgart transplant center (418 grafts), and the transplant center of the Technical University in Munich (330 grafts). Partner grafts were defined as transplantations where both kidneys from a single donor were transplanted in different individuals. We collected donor and recipient characteristics with their respective clinical outcome after transplantation. The local ethics committees authorized the study without a requirement for individual consent. The following inclusion criteria were applied: recipient age of 18 years or older; offer of a kidney-organ from a deceased donor via Eurotransplant; and transplantation of both kidneys from a single donor at the same center. Exclusion criteria were combined organ offers (heart-kidney and pancreas-kidney).

### Outcome

**Figure 1:**
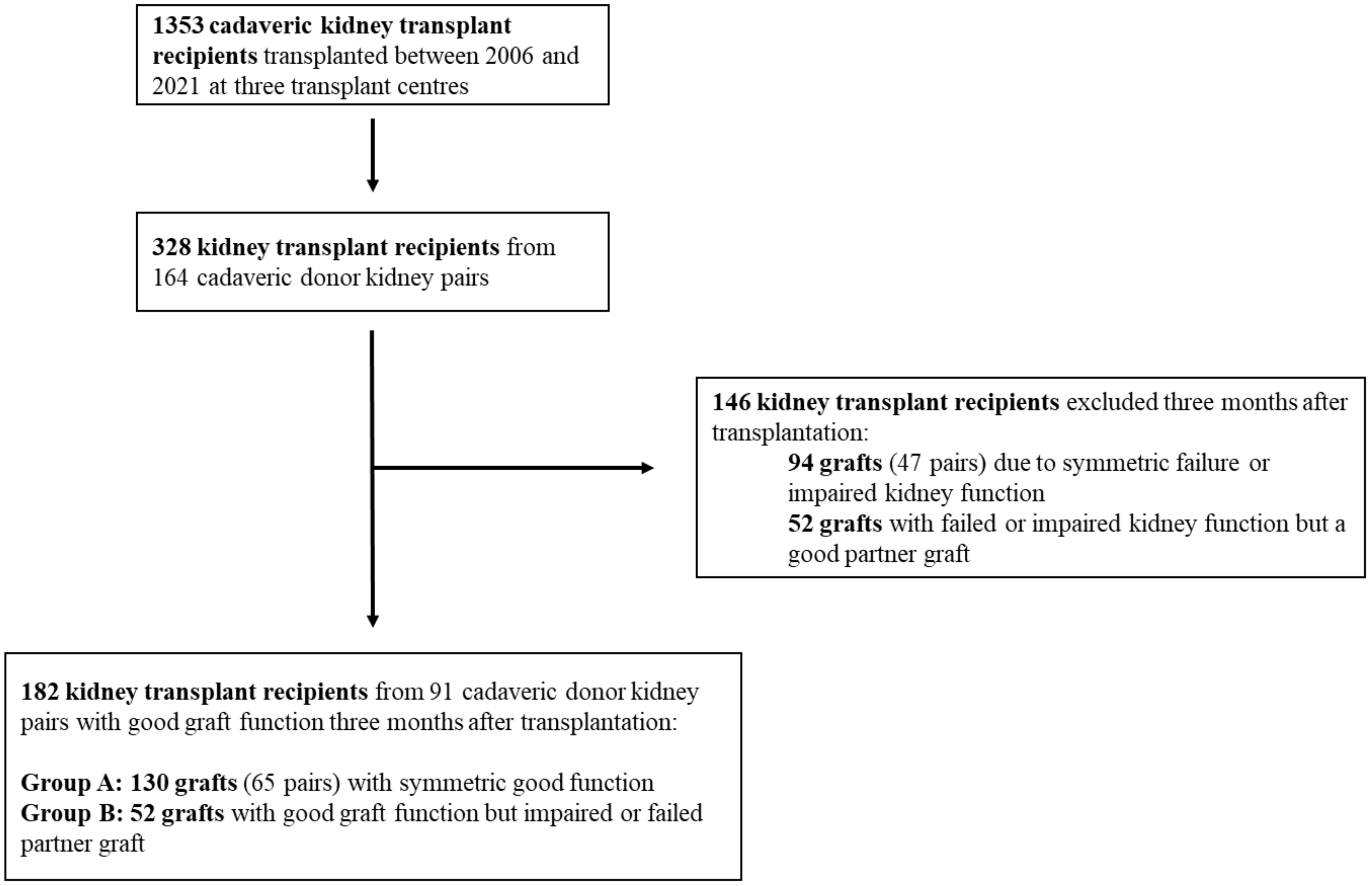
Flowchart of patient selection for statistical analysis. Good graft function: eGFR > 30 mL/min; failed or impaired graft function ≤ 30 mL/min.

The primary outcome was graft survival. We reasoned that donor-related graft function might be most prominent during the early phase after transplantation, whereas recipient and environmental factors mainly bias late graft failure. Therefore, survival analysis was censored at 5 years from transplantation. The CKD-EPI formula was used for calculation of donor eGFR and recipient eGFR after transplantation. A total of 1,353 transplanted grafts were included in the study. From these, we selected 164 kidney pairs (328 grafts). For survival analysis, 146 grafts were excluded because of symmetric failure or impaired function. Groups were analyzed according to concordance of graft function 3 months after transplantation. Group A: good index graft function with symmetric (good) partner graft function; and group B: good index graft function with asymmetric (failed or impaired) partner graft function (see **Figure 1**).

### Statistical analysis

The data collection in the context of the presented project was performed with the help of an electronic database system (Microsoft Excel 2018, Microsoft Germany GmbH, Unterschleißheim). A statistical evaluation was then carried out using RStudio (R team 2021).

There were no missing data for 3-month graft function, graft survival, or 3-month recipient survival. There was less than 1.5% of data missing. Merged multiple imputation was used to compute the mean of all imputed values of each missing value. Paired grafts were identified using a unique identifier, and pairs were sorted and merged to ensure correct pairing. For comparisons of groups, we performed the chi-square test for categorical data, the Mann–Whitney rank test for non-parametric data, and the independent t-test for normal distributed data.

### Survival analysis

The Kaplan–Meier estimator was used to calculate survival probabilities at 3, 12, and 60 months. The expected versus observed graft outcomes were calculated and compared using chi-square tests. The expected counts of symmetrically failed grafts were calculated using the following formula:


$$\text{Both Grafts Failed}=p_{\text{failure}}^{2}\times N_{\text{pairs}}$$

where ${\text{p}}_{\text{failure}}$ is the failure probability at the specified time point, and $N_{\text{pairs}}$ is the total number of pairs. Survival data were reshaped, combined, and filtered to include only data from 3 months after transplant, and censoring was accounted for. A Cox proportional hazards model with frailty for groups was fitted. Survival functions were plotted and compared using the log-rank tests.

### Variable selection and clustered analysis

Univariate generalized estimating equation (GEE) models were employed to calculate odds ratios and confidence intervals for individual variables. For variable selection, we applied Lasso regression, using the GEE model framework, to identify significant predictors. Bootstrapping was used to ensure the stability and reliability of the selected variables. A multivariate GEE model was then fitted on the basis of the previously selected variables. The KDPI was calculated using standard clinical parameters. Descriptive statistics for KDPI and the AUC for 3-month eGFR >30 were generated.

### Data availability

The data and code that support the findings of this study are available from the corresponding authors upon reasonable request.

## Results

### Five-year graft survival based on symmetric versus asymmetric graft function 3 months after transplantation

To assess the impact of donor factors on graft function, we first explored concordance of outcomes for 164 kidney pairs. First, we explored whether outcomes 3, 12, and 60 months after transplantation were donor-dependent, assuming that if the donor plays an important role, then symmetric graft failure would be more frequent than expected for unrelated grafts. The observed and expected frequencies of paired graft failure at the respective time points were (1 versus 2, 3 versus 5, and 11 versus 13; **Figure 1**). When we completed a chi-square test, there was no significant difference between the observed and expected failure rates and observed failure rates were consistently lower than expected. This would argue against a strong role of donor dependent factors for transplant outcome at these time points.

**Figure 2:**
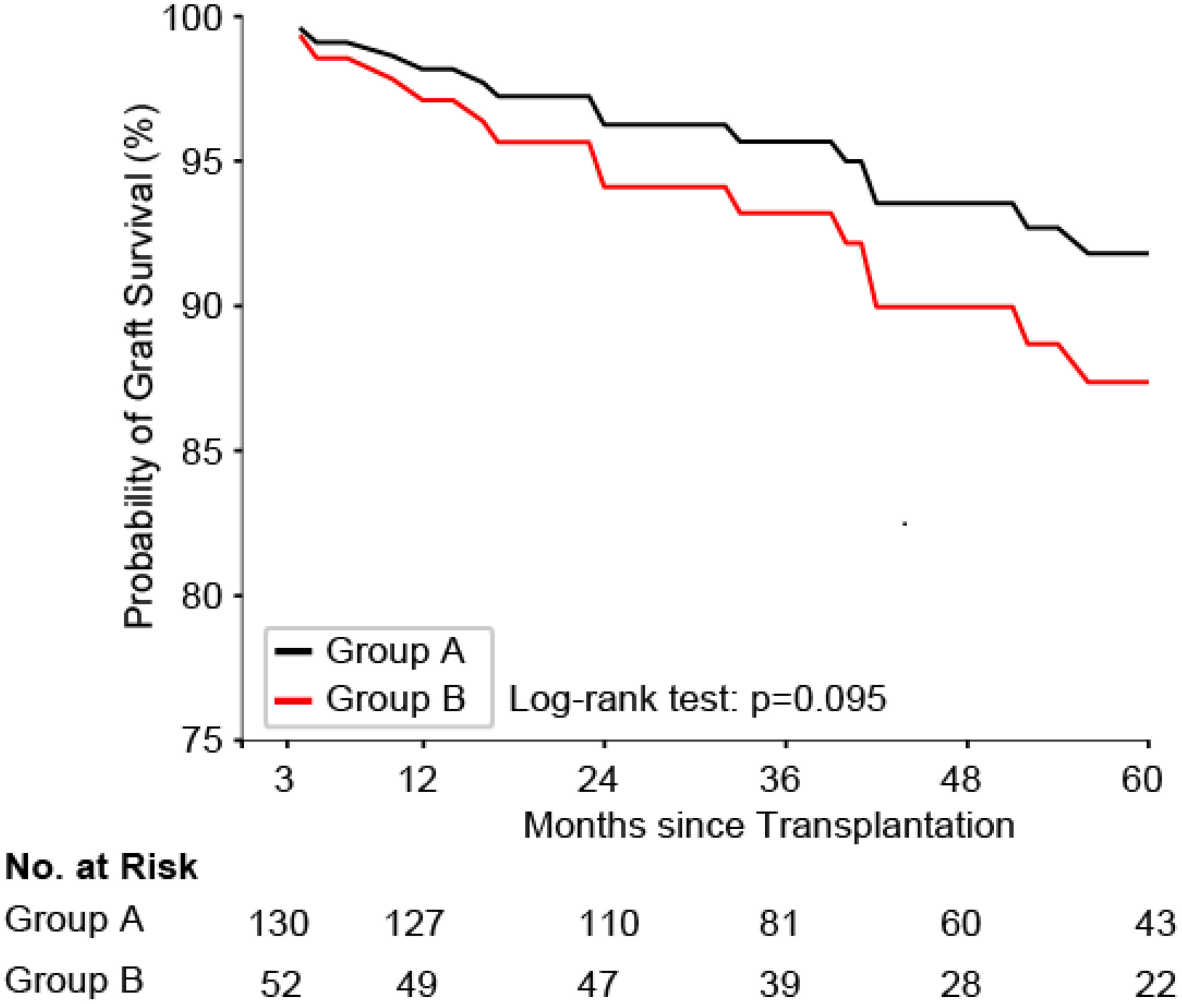
Graft survival probability for group A (symmetric good early graft function) versus group B (asymmetric graft function with good index graft at 3 months from transplantation). Kaplan–Meier survival curves comparing graft survival probabilities between groups over 60 months after transplantation. A trend for higher survival probability of group A (black line) compared to that of group B (red line) is shown, although the difference is not statistically significant (log-rank test, p = 0.095). The analysis accounted for the paired nature of the data using a cox frailty model. The number of grafts at risk in each group at different time points is displayed below the graph.

Second, we explored whether grafts with a good early function differed in their survival rates depending on the partner graft function at 3 months from transplantation. Hence, we compared the survival curves (**Figure 2**) of group A (symmetric good partner graft function at 3 months) versus that of group B (asymmetric graft function at 3 months, good index graft function). Although there was a trend for reduced graft survival in the asymmetric group, this was not significant (log-rank test, p = 0.09). For illustration of the distribution of eGFR differences within pairs at 3 months after transplantation, see a histogramm in **Supplementary Figure 1**. Taken together, our statistical analysis demonstrates the complex interplay of donor, recipient, and periprocedural aspects influencing short-term graft function and long-term graft survival. We show a trend toward more graft failure within 5 years after transplantation in patients with a good short-term graft function but an impaired partner graft function compared to patients with a symmetric good graft function 3 months after transplantation. These results thus provide a rationale for further elucidating specific factors that confer donor-associated risks and could improve graft-offer acceptance decisions in deceased-donor kidney transplantation.

### Comparison of donor and recipient characteristics between groups

To assess whether we could identify specific donor and recipient associated risk factors for graft impairment early after kidney transplantation, we performed a statistical comparison between the two groups (group A: index graft with a eGFR > 30 mL/min 3 months after transplantation, partner graft symmetric; group B: index graft with a eGFR > 30 mL/min 3 months after transplantation, partner graft asymmetric; donor characteristics, **Table 1**). Using this approach, we found that groups differed significantly for the following donor factors: donor age, body mass index (BMI), donor length of stay ≥ 10 days before explantation, and donor eGFR (mL/min). Similarly, groups differed for the recipient factors age, time on dialysis before transplantation, diabetes, ciclosporin, and number of human leucocyte antigen (HLA) mismatches. (recipient characteristics, **Table 2**). Interestingly, we showed a significant reduction of kidney function in patients with an impaired/failed partner graft compared to both (symmetrical) well-functioning grafts (group A: eGFR 94 mL/min versus group B: eGFR 81 mL/min, p < 0.001).

**Table 1:** Donor characteristics.

	Index graft function (eGFR^b^ > 30 mL/min) 3 months after Tx^a^		
	Symmetric partner graft function	Asymmetric partner graft function	P-value
	(N = 130)	(N = 52)	
Modifiable and non-modifiable risk factors			
Sex (male)	62 (48)	28 (54)	0.46
Age (year)	57 [54–59]	69 [67–72]	<0.001
Body-Mass-Index	28 [27–28]	27 [26–28]	0.14
Cause of death			0.65
CVA/stroke	76 (59)	33 (63)	
Other	54 (42)	19 (36)	
Hypertension	75 (58)	36 (69)	0.17
Diabetes	20 (17)	3 (7)	0.13
Length of stay ≥ 10 days	8 (6)	2 (4)	0.02
Donor kidney function			
eGFR^b^ (mL/min)	95 [91–99]	82 [77–88]	<0.001
Diuresis (mL/h)^c^	168 [149–186]	166 [139–193]	0.94

Data provided as N (%, rounded to integers) or median [IQR] based on available data; p-values underlined if < 0.05; ^a^Tx, transplantation; ^b^eGFR, estimated glomerular filtration rate (CKD-EPI, Chronic Kidney Disease Epidemiology Collaboration); ^c^at day of explantation.

**Table 2:** Procedural and recipient characteristics.

	Index graft function (eGFR^b^ > 30 mL/min) 3 months after Tx^a^		
	Symmetric partner graft function	Asymmetric partner graft function	P-value
	(N = 130)	(N = 52)	
Recipient characteristics			
Sex (male)	91 (70)	36 (69)	0.92
Age (year)	55 [53–57]	63 [61–65]	<0.001
Body mass index	25 [24–26]	26 [25–27]	0.21
Time on dialysis before current tx^a^ (years)	7 [5–10]	6 [3–7]	<0.002
PRA^c^ > 5%	21 (16)	5 (9)	0.11
Diabetes	21 (16)	18 (35)	0.01
HLA^d^ - mismatches	4 [3–4]	4 [4–5]	0.006
Graft function and immunosuppression			
Graft function 3 months after tx^a^			
eGFR^b^ (mL/min)	94 (23)	81 (20)	<0.001
Initial graft function			0.94
Primary function	90 (70)	35 (67)	
Delayed graft function	40 (31)	17 (33)	
Ciclosporin	31 (24)	27 (52)	<0.001
Cold ischemia time (h)	12 [11–13]	12 [11–14]	0.98

Data provided as N (%, rounded to integers) or median [IQR] based on available data; p-values underlined if < 0.05; ^a^Tx, transplantation; ^b^eGFR, estimated glomerular filtration rate (CKD-EPI, Chronic Kidney Disease Epidemiology Collaboration); ^c^PRA, panel-reactive-antibody; ^d^HLA, human leucocyte antigen.

### Regression analysis for early graft failure or reduced graft function at 3 months after transplantation

To explore whether clustered analysis after feature selection with LASSO (least absolute shrinkage and selection operator) bootstrap could reveal distinctive donor-associated risk factors for EGL or reduced graft function (eGFR ≤30 mL/min), we analyzed differentiation parameters for each dimension of graft characteristics (donor age, donor-associated modifiable risk factors, donor kidney function, and HLA mismatches), as well as procedural and recipient factors via univariate analysis for their power to separate different early post-transplant outcomes. In the multivariate model based on the selected variables, optimal differentiation yield was obtained for donor age, donor eGFR, and BMI (**Table 3**). Using these criteria, the five-fold validated Area under the curve (AUC) was 0.77. Interestingly, as a comparative analysis, KDPI was calculated (see **Supplementary Table 1**), with an AUC concerning the predictive power of 0.51.

**Table 3:** Regression analysis: graft outcome^a^ 3 months after Tx.

	Univariate analysis			Clustered analysis with GEE^h^ AUC 0.77		
	OR^b^	95% CI^c^	P-value^d^	OR^b^	95% CI^c^	P-value^d^
Donor characteristics						
Age (year)	1.10	1.06–1.13	0.01	1.08	1.05–1.11	0.01
Sex (male)	0.74	0.44–1.24	0.26			
Body mass index	0.96	0.90–1.03	0.03	0.94	0.87–1.01	0.04
eGFR^e^ (mL/min)	0.97	0.96–0.99	<0.01	0.98	0.96–0.99	<0.01
Hypertension	0.99	0.59–1.68	0.26			
Diabetes	1.15	0.58–2.28	0.359			
Cause of death	0.77	0.59–1.02	0.13			
Periprocedural characteristics						
Cold ischemia time (h)	0.98	0.93–1.02	0.02			
Delayed graft function	3.00	2.11–4.26	0.14			
HLA-MM^f^	1.24	1.03–1.49	0.09			
Ciclosporin	1.49	0.92–2.41	0.24			
Recipient characteristics						
Age (years)	1.08	1.05–1.11	0.01			
Sex (male)	0.98	0.62–1.53	0.23			
Body mass index	1.04	0.99–1.09	0.02			
Years on dialysis	0.99	0.98–0.99	<0.01			
PRA^g^ > 5%	1.00	0.99–1.01	<0.01			
Diabetes	1.38	0.84–2.25	0.26			

^a^Outcome: death-censored early graft loss or eGFR ≤ 30 mL/min 3 months after tx (transplantation) of 328 patients; ^b^OR, odds ratio; ^c^confidence interval; ^d^p-values underlined if <0.05; ^e^eGFR, estimated glomerular filtration rate (CKD-EPI) as continuous variable; ^f^HLA-MM, human leukocyte antigen mismatch; ^g^PRA, panel-reactive antibody; ^h^GEE, generalized estimating equations after feature selection with LassoGEE bootstrap. Tx, transplantation.

## Discussion

EGL after kidney transplantation is a disastrous event with far-reaching consequences for the recipient (7). The primary concern of clinicians involved in the kidney organ allocation process should, therefore, be to optimize organ allocation. In this context, donor-based scoring systems have been developed, such as the KDRI and KDPI. However, despite widespread usage in the United States, the score might underestimate the complexity within the organ allocation process, omitting individual aspects such as procedural factors, immunological constellation, or recipient characteristics. Moreover, the KDRI has been suggested to facilitate increased discard rates in the United States indicating a limited usefulness of the donor-based index (7, 8). A Canadian study by Rose et al. suggests a comparable predictive performance of the donor age compared to the full KDRI (9). Similarly, European studies show limitations of the KDRI usage (10, 11, 19). This is in line with our study; the AUC of the KDPI predicting 3-month graft impairment was 0.5.

The question concerning the general significance of donor factors arises. In this regard, ambiguous data exist (4, 12). Therefore, in this study, we investigated the significance of donor factors using the outcome of kidney donor pairs transplanted at two Eurotransplant centers.

First, we hypothesized donor factors to play a crucial role in case of a higher symmetric rate of failed or impaired partner grafts. However, this correlation could not be proved in our study. The observed rate was even lower than expected. Second, we postulated an important donor relevance if 5-year graft survival differed within grafts with good short-term function in dependence of their partner graft function (good symmetric versus impaired asymmetric). No significant difference was shown, however, at least, a trend toward a better graft survival in case of symmetric good partner graft function (p = 0.09). Possibly, the lack of significance is associated with a limited case number. In addition, we showed a significant impaired kidney function 3 months after transplantation of kidney transplant recipients with asymmetric graft function compared to pairs with good symmetric kidney function (81 mL/min versus 94 mL/min, p < 0.001).

Hence, we show donor factors not to be the main criterium in the selection of donor organs; it is a more complex interplay of also recipient and periprocedural factors, in predicting short-term graft function. However, donor factors are part and play an important role. This is in line with several other publications. Traynor et al. discussed a relationship of partner graft function and delayed graft function (13). Gourishankar et al. concluded a similarity in kidney function however a missing link between donor factors and rejection rates (14). This fits our data well.

Meanwhile, the influence of donor factors on graft survival has been tested in larger registry studies. OPTN (Organ Procurement and Transplantation Network) and USRDS (United States Renal Data System) data confirm kidney pairs to have similar outcomes concerning Delayed graft function (DGF). However, regarding long-term outcome, the effect weakens, pointing toward a growing importance of recipients factors (15). Similarly, in the present study, graft failure is significantly increased within the first 6 months after transplantation if the partner graft failed or shows poor function 3 months after transplantation. Afterward, the incidence of graft failure is similar between groups.

Summarizing, in our opinion, application of purely donor-based indices such as the KDRI in the organ allocation process needs further assessment. Statistical analyses of the KDRI within the Dutch registry showed only the minority of differences in 5-year transplantation outcome are explained by donor factors implied in the KDRI (16).

Hence, in the next step, we determined statistically significant donor and recipient factors using univariate analyses within the whole study population detached from the kidney pair approach. Using GEEs after feature selection with LassoGEE bootstrap, we explored transplantation related variables for an optimal differentiation of early graft outcome. This yielded a combination of donor factors (age, baseline eGFR, and BMI) using these criteria, and the 5-fold validated AUC was 0.77.

The outcome after transplantation seems to be a complex interaction of not only donor but also recipient characteristics and periprocedural factors. In addition, the post-transplantation treatment including the interplay of immunosuppressive medication and recipient compliance as well as the prompt anticipation of clinical complications is of immense importance. Relying on solely donor-derived scores might lead to an unjustified high discard rate of possible donor organs (17, 18). This is in line with recent UK and Dutch data that suggest the impact of donor factors on early graft failure to be limited, once a graft was accepted for transplantation (12).

Our study has several limitations. Mainly, we present a limited number of transplant recipients as we only included cases where both partner grafts were transplanted at the same center. Hence, the number of events fulfilling the primary outcome is low, attenuating the statistical power.

In summary, we show a trend toward an increased risk of graft failure of grafts with good graft function 3 months after transplantation within 5 years after transplantation, if the partner graft shows a poor graft function or failure at 3 months after transplantation. This suggests a relevant effect of donor factors predicting especially the early success after transplantation. However, without statistical significance concerning long-term graft survival, the present study does not justify to rely transplant acceptance decision only on donor factors. Within donor risk factors, present study once again proves donor age to represent the most important predictor.

On the basis of the present data, we suggest to further focus on elaborating the most important donor factors and to translate them into a patient- and clinician-friendly tool to support our organ acceptance strategy. Nevertheless, the final decision should remain with the individual clinician weighing off the complex interplay of not only the donor and the recipient but also periprocedural aspects.

## Data Availability

The raw data supporting the conclusions of this article will be made available by the authors, without undue reservation.
