# Supplementary material for: Impact of deceased-donor characteristics on early graft function: outcome of kidney donor pairs accepted for transplantation
Source: Front Immunol. 2024 Oct 8;15:1303746. doi: 10.3389/fimmu.2024.1303746 (PMC11493709; doi:10.3389/fimmu.2024.1303746)
Supplement: Supplementary file 1 [file DataSheet1.docx]

**Impact of deceased-donor characteristics on early graft function:**

**Outcomes of kidney donor pairs accepted for transplantation**

**Supplementary material & methods**

**Supplemental Figures**

**Supplemental Figure 1:**


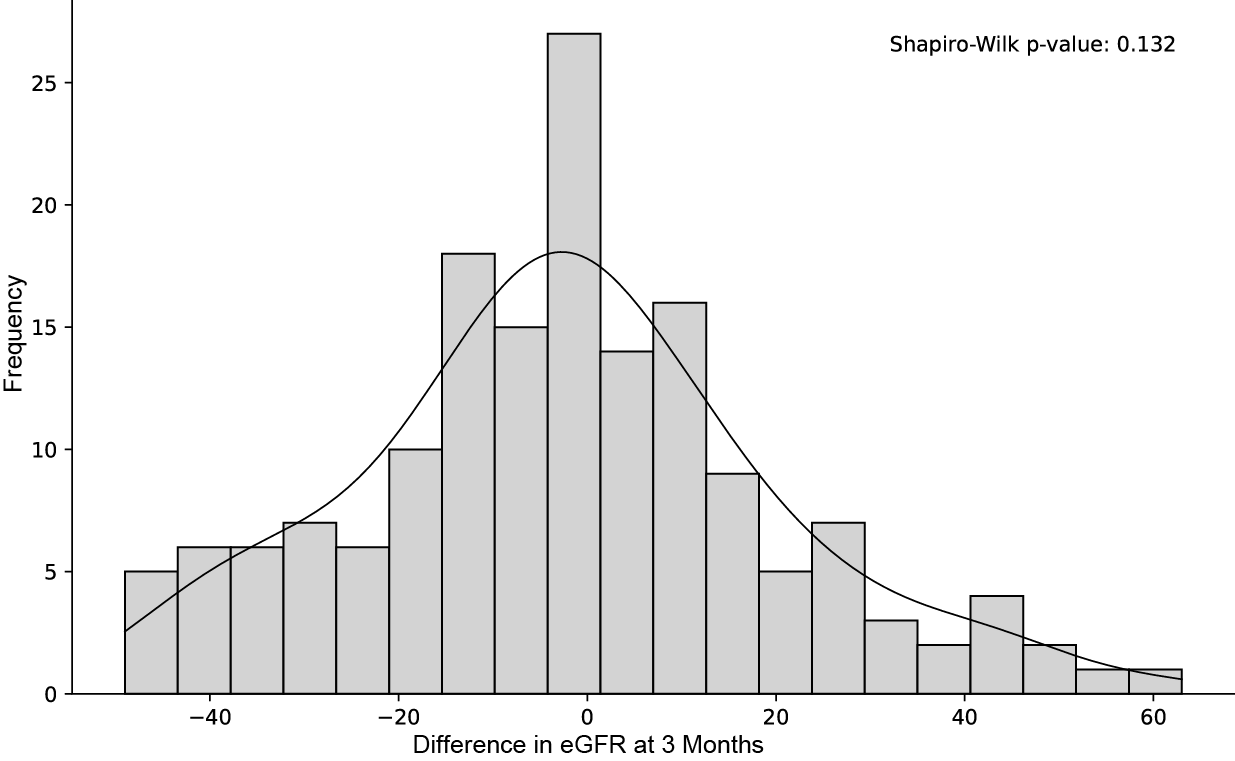


**Histogramm of eGFR difference within pairs.** Frequency of differences in eGFR at three months from transplantation. A density curve was estimated using kernel density estimation (KDE). Shapiro-Wilk test for normality was conducted to determine whether the distribution of graft survival months follows a normal distribution. P is 0.13, this supports the assumption that the data is normally distributed.

**Supplemental Table 1. KDPI**

| **Donor Factors** | **N=328** |
| --- | --- |
| Age, years | 65 (13) |
| Height, cm | 172 (9) |
| Weight, kg | 80 (15) |
| Hypertension | 188 (63) |
| Diabetes | 40 (14) |
| Cause of Death |  |
| Cerebral infarction | 22 (7) |
| Bleeding | 194 (59) |
| Trauma | 22 (7) |
| Others | 90 (27) |
| Creatinine at explantation, mg/dl | 1.2 (1.0) |
| **KDPI, mean (SD)** | **83 (22)** |
| **KDPI-AUC** | **0.51** |

**KDPI of all 328 patients.** Data is given in mean (SD) or number (%). AUC for the prediction of a three-months eGFR > 30 ml/min
